# *PiggyBac* Transposon-Mediated Transgenesis in the Pacific Oyster (*Crassostrea gigas*) – First Time in Mollusks

**DOI:** 10.3389/fphys.2018.00811

**Published:** 2018-07-16

**Authors:** Jun Chen, Changlu Wu, Baolu Zhang, Zhongqiang Cai, Lei Wei, Zhuang Li, Guangbin Li, Ting Guo, Yongchuan Li, Wen Guo, Xiaotong Wang

**Affiliations:** ^1^School of Agriculture, Ludong University, Yantai, China; ^2^Consultation Center, State Oceanic Administration, Beijing, China; ^3^Changdao Enhancement and Experiment Station, Chinese Academy of Fishery Sciences, Yantai, China; ^4^Center for Mollusc Study and Development, Marine Biology Institute of Shandong Province, Qingdao, China

**Keywords:** Pacific oyster, transgenesis, *piggyBac* transposons, sperm-mediated gene transfer, electroporation

## Abstract

As an effective method of transgenesis, the plasmid of *PiggyBac* transposon containing GFP (*PiggyBac*) transposon system has been widely used in various organisms but not yet in mollusks. In this work, *piggyBac* containing green fluorescent protein (GFP) was transferred into the Pacific oyster (*Crassostrea gigas*) by sperm-mediated gene transfer with or without electroporation. Fluorescent larvae were then observed and isolated under an inverted fluorescence microscope, and insertion of *piggyBac* was tested by polymerase chain reaction (PCR) using genomic DNA as template. Oyster larvae with green fluorescence were observed after transgenesis with or without electroporation, but electroporation increased the efficiency of sperm-mediated transgenesis. Subsequently, the recombinant *piggyBac* plasmid containing *gGH* (*piggyBac-gGH*) containing GFP and a growth hormone gene from orange-spotted grouper (*gGH*) was transferred into oysters using sperm mediation with electroporation, and fluorescent larvae were observed and isolated. The insertion of *piggyBac-gGH* was tested by PCR and genome walking analysis. PCR analysis indicated that *piggyBac-gGH* was transferred into oyster larvae; genome walking analysis further showed the detailed location where *piggyBac-gGH* was inserted in the oyster genome. This is the first time that *piggyBac* transposon-mediated transgenesis has been applied in mollusks.

## Introduction

Many methods can be employed to achieve genetic transformation, including viral vectors, non-viral vectors and transposons. Among these relatively effective methods of transgenesis, transposons have the ability to integrate into chromosomes, and gene expression from transposons is permanent (Ivics and Izsvak, 2006). Transposons are mobile elements that can transpose between vectors and a target genome. The original *piggyBac* transposon was first identified and isolated from the genome of the cabbage looper moth, *Trichoplusia ni*, in the late 1980s (Cary et al., 1989). Later,

other *piggyBac*-like transposons were identified in different insect species (Wu et al., 2011; Depra et al., 2012; Sormacheva et al., 2012), frogs (Lam et al., 1996; Leaver, 2001), fish (Cocca et al., 2011; Costa et al., 2013), and mammals (Jurka, 2000; Sarkar et al., 2003). The original *piggyBac* element is approximately 2.4 kb, with identical 13 bp inverted terminal repeats and additional asymmetric 19 bp internal repeats (Elick et al., 1997; Li et al., 2001, 2005). Previous data have shown that the original *piggyBac* transposon can effectively transfer up to 9.1 kb of transgene sequence into chromosomes with the help of the *piggyBac* transposase enzyme from a separate plasmid (Ding et al., 2005). *PiggyBac* allows “cut and paste” transfer of genes of interest between inverted terminal repeats and internal repeats into a host genome at TTAA nucleotide elements (Fraser et al., 1996; Bauser et al., 1999; O’Brochta et al., 2003; Wu and Burgess, 2004). *PiggyBac* transposon can insert into gene regions and cause mutagenesis, which contributes to infer function of the inserted gene (Ding et al., 2005). Furthermore, *PiggyBac* transposon can introduce exogenous genes into the genome for constructing transgenic organisms or testing gene function (Yusa, 2015). Thus, *piggyBac* transposons have been successfully used as a promising tool for basic genetic studies (Wang et al., 2008; Kaji et al., 2009; Woltjen et al., 2009; Yusa et al., 2009), gene therapies (Carlson et al., 2005) and construction of transgenic animals (Ding et al., 2005). Studies have also shown that the original *piggyBac* transposon has a broad host spectrum ranging from yeast to mammals, and this mobile element has been widely used for a variety of applications in a diverse range of organisms. However, the *piggyBac* transposition system has not yet been utilized or characterized in mollusks.

It has been reported that exogenous DNA can stick to spermatozoa and then be carried into an egg via fertilization. This method of sperm mediation is widely used for gene transfer (Parrington et al., 2011). Previous research has also investigated approaches for increasing the proportion of spermatozoa carrying exogenous DNA to improve the efficiency of transgenesis. One effective method is electroporation of spermatozoa during transformation, which increases the capacity of DNA bound by sperm in fish (Patil and Khoo, 1996) and mollusks (Tsai, 2000).

Pacific oyster is one of the most economically important cultivated oysters in the world. Genome sequencing of Pacific oyster has been completed (Zhang et al., 2012). However, the functions of most Pacific oyster genes are unknown due to a lack of effective research methods, such as transgenesis technology. In this work, we demonstrate that the *piggyBac* transposon system is suitable for Pacific oyster transgenesis.

## Materials and Methods

### Plasmid Construction

The donor plasmid *piggyBac* Dual Promoter and helper plasmid HA-mPB were constructed by Applied Biosystems. *PiggyBac* Dual Promoter carries the CMV promoter upstream of a MCS. Downstream of the expression cassette is an EF1alpha promoter driving the expression of a GFP+Puro marker. The entire cassette is flanked by genomic insulator elements to stabilize expression and *piggyBac* ITR for mobilization and integration. The helper plasmid HA-mPB contains the mPB transposase coding sequence in a pcDNA3-Kz-HA backbone, and it features a 5′ HS4 insulator to support robust transcription from the rPolr2A promoter.

Sequence encoding the mature GH peptide of orange-spotted grouper was amplified by primers GH-F and GH-R, with EcoRI at the 5′ end and BamHI at the 3′ end, using grouper pituitary cDNA as template. Then, the resultant GH fusion gene was digested with EcoRI and BamHI, purified and ligated into the EcoRI and BamHI sites of plasmid *piggyBac* to construct *piggyBac-gGH.*

### Transfection of *piggyBac* Into Pacific Oysters

Five hundred μL of semen sampled from mature male Pacific oysters was collected into conical polystyrene sample cups, and then diluted to a concentration of 10^5^ sperm/mL. Then, a combination of “helper-independent” transposase (15 μg) and transposon (30 μg) were added to the semen, and the mixture was incubated for 5 min at 20°C. For sperm-mediated gene transfer, approximately 10^4^ eggs collected from mature female Pacific oysters were added to the semen-plasmid mixture, and then the mixture was incubated for 5 min. Finally, seawater was added into the mixture to activate fertilization.

**FIGURE 1:**
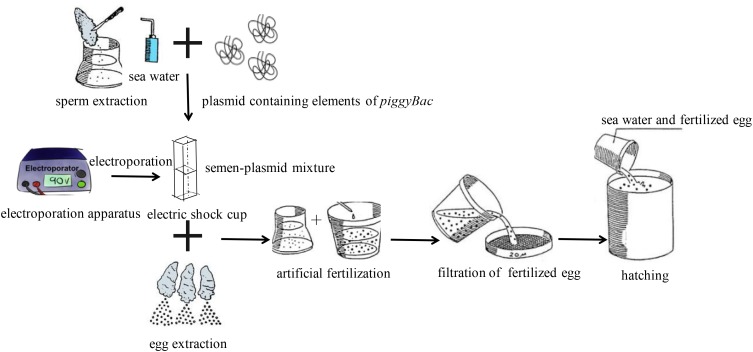
Schematic diagram of procedures for transfection of *piggyBac* into Pacific oyster.

For electroporation-mediated gene transfer, the semen-plasmid mixture was transferred into an electric shock cup, and then treated five times with an electroporation apparatus (Scientz, Wuhan, China) with the following parameters: resistance, 100 Ω; voltage, 300 V; capacitance, 100 μF. And each electric pulse duration was 20 ms, and the interval between two pulses was 100 ms. Then, fertilization was carried out as described above. Finally, fertilized ova were cultured in a 10-L tank at 20°C. During transgenesis, the same treatment was performed without plasmid as a negative control. All treatments were repeated three times, and three individual experiments were performed. Additionally, Schematic diagram of procedures for transfection of *piggyBac* into Pacific Oysters was shown in **Figure 1**.

### Microscopic Observation of Fluorescent Oyster Larvae and Calculation of Transfection Efficiency and Relative Transfection Efficiency

Survival rates of oyster larvae were initially estimated by counting the density of oyster larvae using an optical microscope 0 and 72 h after fertilization. Oyster larvae transfected with both donor and helper plasmids were observed at 72 h post-transfection using a fluorescence microscope (IX71, Olympus, Japan), under which the expressed fluorescence appeared green. Fluorescent oyster larvae were photographed, counted and isolated.

Next, the efficiency of transfection (%) and relative efficiency of transfection (%) were calculated as below. Efficiency of transfection (%) = Number of larvae expressing fluorescence/Total number of fertilized eggs; relative efficiency of transfection (%) = Number of larvae expressing fluorescence/Total number of larvae at 72 h.

### PCR and Sequence Analysis

QIAamp^®^ DNA Micro Kit (Qiagen, United States) was used for trace DNA extraction from oyster larvae. Primers specific for GFP, GH, GH-GFP and the sequence downstream of GFP were used to confirm that *piggyBac-GFP* and *piggyBac-gGH* were successfully inserted and to identify the insertion site within the Pacific oyster genome (primers are shown in **Table 1**). Briefly, PCR amplification for GFP, GH, or GH-GFP sequences was conducted with 35 cycles of 95°C for 20 s, 60°C/60°C/56°C for 20 s, and 72°C for 50 s/1 min/1 min 20 s. PCR products were analyzed by agarose gel electrophoresis and sequencing. Additionally, the oyster genomic insertion site was analyzed using a genome walking kit (Takara, Japan). All primers are shown in **Table 1**.

**Table 1:** Primers used in this study.

Primer	Sequence (5′→3′)	Purpose
GFP-F	TGACCAACAAGATGAAGAGCACC	Cloningof GFP gene
GFP-R	TCCACGTCACCGCATGTTAGAAGA	Cloning of GFP gene
GH-F	CGGAATTCATGGACCGA GTCGTCCTCCTG	Cloning of GH gene
GH-R	CGGGATCCCTACAGGGTA CAGTTGGCCTC	Cloning of GH gene
GH-GFP-F	AACTGCTGGCTTGTTTCA	Cloning of GH-GFP gene
GH-GFP-R	CGATGCGGGTGTTGGTGT	Cloning of GH-GFP gene
SP1	ATTCCACACAACATACGAGCCG	Genome walking
SP2	CTGGGGTGCCTAATGAGTGAGC	Genome walking
SP3	GGGAGAGGCGGTTTGCGTATTG	Genome walking


*The base sequences of restriction enzyme cutting sites are underlined.*

### Statistical Analysis

All data were analyzed using SPSS 20.0 software (Chicago, IL, United States) with one-way variance (ANOVA) followed by Student–Newman–Keuls multiple comparisons test. Differences were considered statistically significant when *p* < 0.05.

## Results

### Construction of *piggyBac-gGH*

**FIGURE 2:**
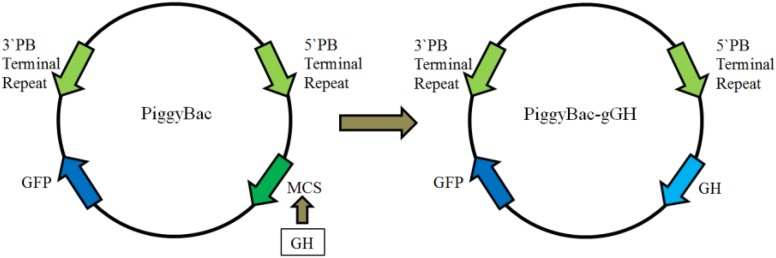
Construction of plasmid *piggyBac-gGH.* The left is the plasmid *PiggyBac*, the right the recombinant plasmid *piggyBac-gGH*. PB, *piggyBac*; GFP, green fluorescent protein; MCS, multiple clone sites; gGH, growth hormone gene of orange-spotted grouper.

As shown in **Figure 2**, a fusion gene encoding the mature *GH* peptide of orange-spotted grouper was subcloned upstream of *GFP* in the donor plasmid *PiggyBac*, producing the recombinant plasmid *piggyBac-gGH*.

### Observation of Fluorescent Larvae and Efficiency of Transfection

**FIGURE 3:**
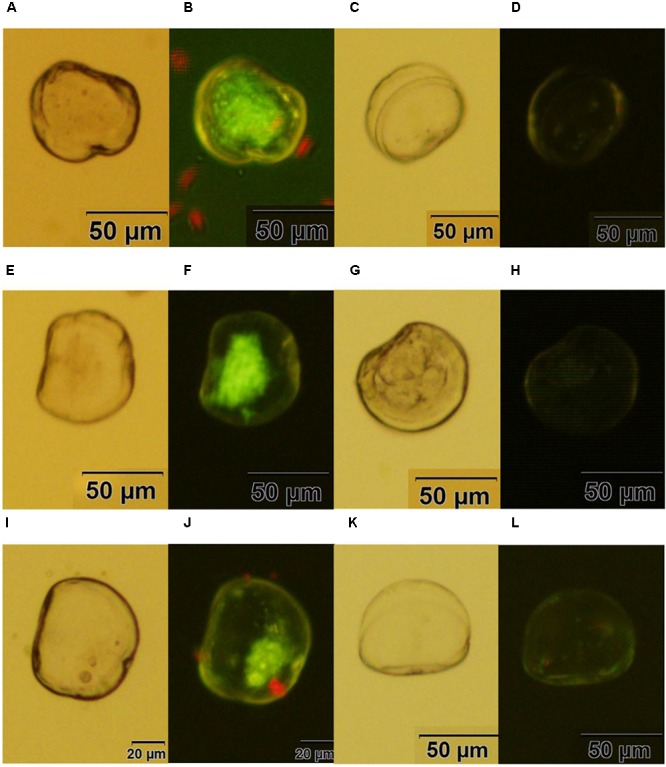
Transgenic oyster larvae and controls. **(A,B)** Oyster larvae transfected with *piggyBac* using sperm mediation without electroporation under white light or green fluorescence; **(C,D)** non-transfected oyster larvae as a control for **(A,B)**; **(E,F)** oyster larvae transfected with piggyBac using sperm mediation with electroporation under white light or green fluorescence; **(G,H)** non-transfected oyster larvae as a control for **(E,F)**; **(I,J)** oyster larvae transfected with *piggyBac-gGH* using sperm mediation with electroporation under white light or green fluorescence; **(K,L)** non-transfected oyster larvae as a control for **(I,J)** Red spots are the algae *Isochrysis galbana* used as food for oyster larvae.

Fluorescent oyster larvae transfected with helper and donor plasmids were observed and counted using a confocal microscope, and then were isolated 72 h after transfection. As shown in **Figure 3**, Pacific oyster larvae with green fluorescence were observed after transformation with *piggyBac* without electroporation (**Figure 3B**), with electroporation (**Figure 3F**) or with *piggyBac-gGH w*ith electroporation (**Figure 3J**), while green fluorescence was not observed in the control group (**Figures 3D,H,L**).

**FIGURE 4:**
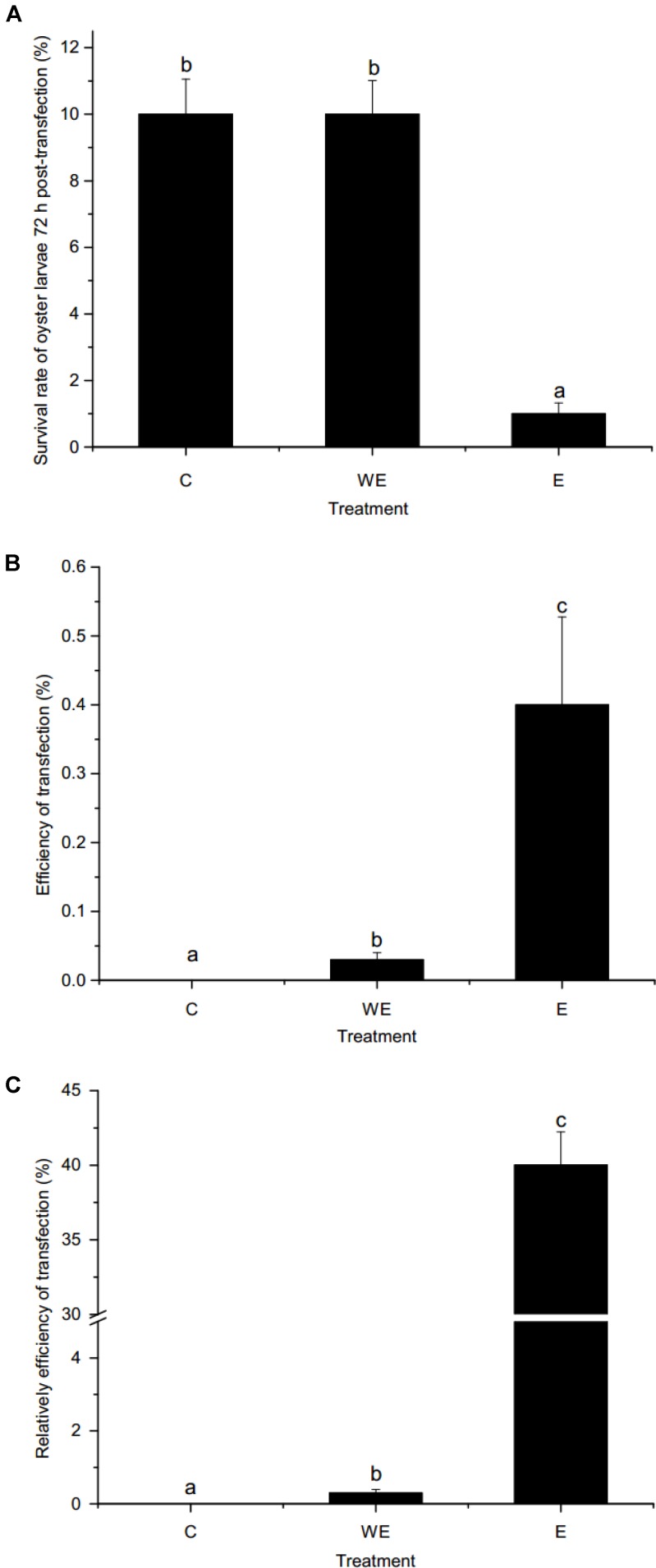
Survival rate and transfection efficiency using *piggyBac* system in oyster larvae. **(A)** Survival rate of oyster larvae 72 h post-transfection; **(B)** efficiency of transfection; **(C)** relative efficiency of transfection. C, non-transfected control group. WE, sperm-mediated transfection without electroporation. E, sperm-mediated transfection with electroporation. Data are shown as the means ± SEM (standard error of mean, SEM), *n* = 3. Different letters indicate statistically significant differences (*p* < 0.05).

As shown in **Figure 4A**, the survival rates of oyster larvae were approximately 10 and 1% after gene transfer of *piggyBac* without or with electroporation, respectively. The survival rate of the electroporation group was significantly lower than that of the control group or the group without electroporation (*p* < 0.01). However, the results (**Figure 4B**) showed that the efficiency of *piggyBac* transfection with electroporation (0.4%) was significantly higher than without electroporation (0.03%). In addition, the relative efficiency of *piggyBac* transfection with electroporation was dramatically higher than transfection without electroporation (40 vs. 0.3%, **Figure 4C**).

### Characterization of Exogenous Gene Insertion

**FIGURE 5:**
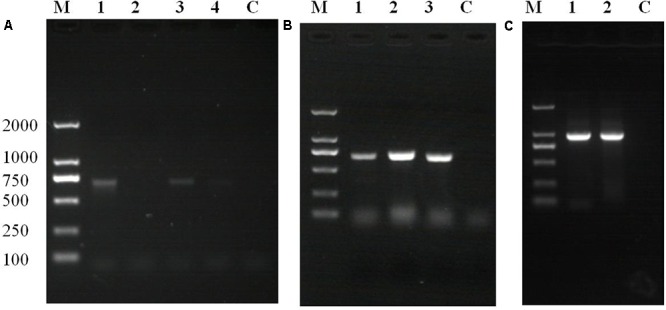
Polymerase chain reaction (PCR) amplification of target genes using the transgenic oyster genome as template. **(A)** GFP gene products amplified from oyster larvae transformed with *piggyBac*: lane M, marker; lane 1, amplification of GFP gene using fluorescent larvae from transgenesis experiment without electroporation; lane 2, amplification of GFP gene using non-fluorescent larvae from transgenesis experiment without electroporation; lane 3, amplification of GFP gene using fluorescent oyster larvae in transgenesis experiment with electroporation; lane 4, amplification of GFP gene using non-fluorescent larvae from transgenesis experiment with electroporation; lane C, control. **(B)** Gene products amplified from oyster larvae transformed with *piggyBac* or *piggyBac-gGH* with electroporation: lane M, marker; lane 1, amplification of GFP gene using fluorescent larvae transfected with *piggyBa*c; lane 2, amplification of GFP gene using fluorescent larvae transfected with *piggyBac-gGH*; lane 3, amplification of GH gene using fluorescent larvae transfected with *piggyBac-gGH*; lane C, control. **(C)** Gene products covering partial sequences of GFP and GH genes amplified from oyster larvae transformed with *piggyB*ac-*gGH* with electroporation: lane M, marker; lanes 1 and 2, amplification of segments covering GH and GFP genes using genome walking; lane C, control.

**FIGURE 6:**
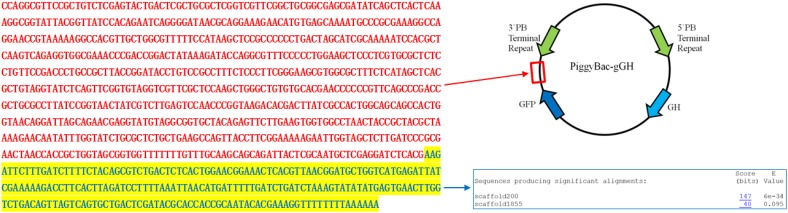
Sequence obtained by genome walking using genomic DNA from transgenic oyster larvae as template. The sequence in red corresponds to partial vector sequence downstream of the GFP gene in *piggyBac-gGH*, and sequence in blue aligns with the scaffold200 in Pacific oyster genome (http://www.oysterdb.com/FrontToolsAction.do?method=blastInit).

Polymerase chain reaction and agarose gel electrophoresis detected the target gene segment in fluorescent oyster larvae, but not in non-fluorescent oyster larvae. As shown in **Figure 5A**, the GFP gene was detected in oyster larvae transfected with *piggyBac.* Both the GFP and GH genes were amplified from genomic DNA of oyster larvae transfected with *piggyBac-gGH* (**Figure 5B**), and sequencing results showed that their sequences matched the appropriate gene sequences in *piggyBac-gGH* (**Supplementary Figures S1**, **S2**). Meanwhile, segments covering partial sequences of GFP and GH were amplified using GH-GFP-F and GH-GFP-R as primers, which indicated that the GFP and GH genes were inserted into the oyster genome together (**Figure 5C** and **Supplementary Figure S3**). Additionally, the genomic insertion site of *piggyBac-gGH* was identified using the method of genome walking, and as shown in **Figure 6**, GFP-GH had been inserted into Scaffold200 of Pacific oyster genome at the sequence “AAGA.”

## Discussion

Sperm-mediated transformation in mollusks has been reported in previous studies with or without electroporation (Tsai et al., 1997; Guerra et al., 2005; Guerra and Esponda, 2006), and CRISPR/Cas9-mediated transformation has also been utilized (Perry and Henry, 2015). In this work, our transgenesis of Pacific oysters with the *piggyBac* transposon system is the first time this system has been used in mollusks.

Our study showed that transgenic fluorescent oyster larvae can be acquired with the *piggyBac* transposon system, and PCR amplification of target genes from the genomes of transgenic fluorescent oyster larvae indicated that exogenous genes were integrated into Pacific oyster genomes. Genome walking is commonly used to identify regions upstream and downstream from known DNA sequences (Rishi et al., 2004; Leoni et al., 2008, 2011; Shapter and Waters, 2014). In this study, the DNA sequence of unknown genomic regions flanking the *piggyBac-gGH* insertion were determined by genome walking, then these sequences were aligned with the oyster genome sequence to determine the insertion site. These results further showed that *piggyBac* transposons can mediate integration of exogenous genes into the oyster genome. However, the insertion site of *piggyBac-gGH* in this study was different from previous studies, which reported that *piggyBac* insertions showed a high preference for genomic TTAA nucleotide elements (Fraser et al., 1995; Balu et al., 2005; Ding et al., 2005; Su et al., 2012; Yusa, 2015).

In this study, the efficiency of transfection using electroporation in Pacific oysters was approximately 0.4%, which is similar to reported rates of 0.1–10% in insects (Fraser, 2012); electroporation of the mixture of spermatozoa and transposase–transposon vectors resulted in about 10-fold higher efficiency of transgenesis than without electroporation, in agreement with earlier research (Patil and Khoo, 1996; Tsai, 2000). These results indicated that electroporation of the mixture of spermatozoa and vectors increased the efficiency of transgenesis. However, data in this study revealed that survival rate of Pacific oysters was decreased by electroporation prior to gene transfer, maybe because spermatozoa were damaged by the electrical impulse.

In addition, the present work would support a new method, *piggyBac* transposon system, for the research fields of genome and gene function in oyster. Firstly, some genes related with economic importance, such as resistance gene, growth hormone gene, or glycogenesis gene, could be introduced into oyster genome by *piggyBac* transposon system to improve the quality of oyster. Moreover, the genes with unknown function also could be transferred into oyster genome and their physiological functions could be deduced according to the phenotype of the transgenic oysters.

## Conclusion

This study demonstrated that the *piggyBac* transposon system can be used for transgenesis of Pacific oysters. This work provides a new chance for studying the gene function of mollusks. And our further research will focus on enhancing the transfection efficiency of Pacific oysters.

## Author Contributions

XW and BZ conceived and designed the experiments. JC, CW, ZC, LW, and ZL performed the experiments. GL, TG, and YL analyzed the data. WG contributed reagents, materials, analysis tools. XW, JC, and CW wrote the paper. All authors reviewed the manuscript.

## Conflict of Interest Statement

The authors declare that the research was conducted in the absence of any commercial or financial relationships that could be construed as a potential conflict of interest.

## Supplementary Material

FIGURE S1 Sequence alignment between *piggyBac* sequence and GFP segment amplified using the genome of the oyster transfected with *piggyBac* as template (PCR amplification using primers GFP-F and GFP-R).

FIGURE S2 Sequence alignment between *piggyBac-gGH* sequence and GH segment amplified using the genome of the oyster transfected with *piggyBac-gGH* as template (PCR amplification using primers GH-F and GH-R).

FIGURE S3 Sequence alignment between *piggyBac-gGH* sequence and GH-GFP segment amplified using the genome of the oyster transfected with *piggyBac-gGH* as template (PCR amplification using primers GH-GFP-F and GH-GFP-R). The sequence at upstream of the first red arrow is GH segment, that between the two red arrows is the partial plasmid sequence, and that at downstream of the second red arrow is GFP segment.

## Abbreviations

CMV: human cytomegalovirus
GFP: green fluorescent protein
*gGH*: a growth hormone gene from orange-spotted grouper
*MCS*: multiple cloning site
*PCR*: polymerase chain reaction
**piggyBac**: the plasmid of *PiggyBac* transposon containing GFP
*piggyBac-gGH*: the recombinant *piggyBac* plasmid containing *gGH*
