# Supplementary material for: PiggyBac Transposon-Mediated Transgenesis in the Pacific Oyster (Crassostrea gigas) – First Time in Mollusks
Source: Front Physiol. 2018 Jul 16;9:811. doi: 10.3389/fphys.2018.00811 (PMC6054966; doi:10.3389/fphys.2018.00811)
Supplement: FIGURE S1 — Sequence alignment between piggyBac sequence and GFP segment amplified using the genome of the oyster transfected with piggyBac as template (PCR amplification using primers GFP-F and GFP-R). [file Image_1.PDF]

Figure S1.

```
GFP-cds PiggyBac             CGGCGGAGAGGGCACCCCCAAGCAGGGCCGCATGACCAACAAGATGAAGAGCACCAAAGGCGCCCTGACCTTCAGCCCCTACCTGCTGAGCCACGTGATG   2500
GFP-cds Transgenic oyster    ...........................................................TAGGGCTGAC.TTCG..CCCTACCTGCTGAGCCACGTGATG     38
Consensus                                                                                 g  ctgac ttc   ccctacctgctgagccacgtgatg

GFP-cds PiggyBac             GGCTACGGCTTCTACCACTTCGGCACCTACCCCAGCGGCTACGAGAACCCCTTCCTGCACGCCATCAACAACGGCGGCTACACCAACACCCGCATCGAGA   2600
GFP-cds Transgenic oyster    GGCTACGGCTTCTACCACTTCGGCACCTACCCCAGCGGCTACGAGAACCCCTTCCTGCACGCCATCAACAACGGCGGCTACACCAACACCCGCATCGAGA    138
Consensus                    ggctacggcttctaccacttcggcacctaccccagcggctacgagaaccccttcctgcacgccatcaacaacggcggctacaccaacacccgcatcgaga

GFP-cds PiggyBac             AGTACGAGGACGGCGGCGTGCTGCACGTGAGCTTCAGCTACCGCTACGAGGCCGGCCGCGTGATCGGCGACTTCAAGGTGGTGGGCACCGGCTTCCCCGA   2700
GFP-cds Transgenic oyster    AGTACGAGGACGGCGGCGTGCTGCACGTGAGCTTCAGCTACCGCTACGAGGCCGGCCGCGTGATCGGCGACTTCAAGGTGGTGGGCACCGGCTTCCCCGA    238
Consensus                    agtacgaggacggcggcgtgctgcacgtgagcttcagctaccgctacgaggccggccgcgtgatcggcgacttcaaggtggtgggcaccggcttccccga

GFP-cds PiggyBac             GGACAGCGTGATCTTCACCGACAAGATCATCCGCAGCAACGCCACCGTGGAGCACCTGCACCCCATGGGCGATAACGTGCTGGTGGGCAGCTTCGCCCGC   2800
GFP-cds Transgenic oyster    GGACAGCGTGATCTTCACCGACAAGATCATCCGCAGCAACGCCACCGTGGAGCACCTGCACCCCATGGGCGATAACGTGCTGGTGGGCAGCTTCGCCCGC    338
Consensus                    ggacagcgtgatcttcaccgacaagatcatccgcagcaacgccaccgtggagcacctgcaccccatgggcgataacgtgctggtgggcagcttcgcccgc

GFP-cds PiggyBac             ACCTTCAGCCTGCGCGACGGCGGCTACTACAGCTTCGTGGTGGACAGCCACATGCACTTCAAGAGCGCCATCCACCCCAGCATCCTGCAGAACGGGGGCC   2900
GFP-cds Transgenic oyster    ACCTTCAGCCTGCGCGACGGCGGCTACTACAGCTTCGTGGTGGACAGCCACATGCACTTCAAGAGCGCCATCCACCCCAGCATCCTGCAGAACGGGGGCC    438
Consensus                    accttcagcctgcgcgacggcggctactacagcttcgtggtggacagccacatgcacttcaagagcgccatccaccccagcatcctgcagaacgggggcc

GFP-cds PiggyBac             CCATGTTCGCCTTCCGCCGCGTGGAGGAGCTGCACAGCAACACCGAGCTGGGCATCGTGGAGTACCAGCACGCCTTCAAGACCCCCATCGCCTTCGCCAG   3000
GFP-cds Transgenic oyster    CCATGTTCGCCTTCCGCCGCGTGGAGGAGCTGCACAGCAACACCGAGCTGGGCATCGTGGAGTACCAGCACGCCTTCAAGACCCCCATCGCCTTCGCCAG    538
Consensus                    ccatgttcgccttccgccgcgtggaggagctgcacagcaacaccgagctgggcatcgtggagtaccagcacgccttcaagacccccatcgccttcgccag

GFP-cds PiggyBac             ATCCCGCGCTCAGTCGTCCAATTCTGCCGTGGACGGCACCGCCGGACCCGGCTCCACCGGATCTCGCGAGGGCAGAGGAAGTCTTCTAACATGCGGTGAC   3100
GFP-cds Transgenic oyster    ATCCCGCGCTCAGTCGTCCAATTCTGCCGTGGACGGCACCGCCGGACCCGGCTCCACCGGATCTCGCGAGGGCAGAGGAAGTCTTCTAACTGGGGG....    634
Consensus                    atcccgcgctcagtcgtccaattctgccgtggacggcaccgccggacccggctccaccggatctcgcgagggcagaggaagtcttctaac  g gg

GFP-cds PiggyBac             GTGGAGGAGAATCCCGGCCCTATGACCGAGTACAAGCCCACGGTGCGCCTCGCCACCCGCGACGACGTCCCCAGGGCCGTACGCACCCTCGCCGCCGCGT   3200
GFP-cds Transgenic oyster    ....................................................................................................    634
Consensus
```
