# Supplementary material for: PiggyBac Transposon-Mediated Transgenesis in the Pacific Oyster (Crassostrea gigas) – First Time in Mollusks
Source: Front Physiol. 2018 Jul 16;9:811. doi: 10.3389/fphys.2018.00811 (PMC6054966; doi:10.3389/fphys.2018.00811)
Supplement: FIGURE S2 — Sequence alignment between piggyBac-gGH sequence and GH segment amplified using the genome of the oyster transfected with piggyBac-gGH as template (PCR amplification using primers GH-F and GH-R). [file Image_2.PDF]

Figure S2.

```
GH-cds PiggyBac-gGH        AGTCGTCCTCCTGCTGTCAGTAGTGTCTCTGGGTGTTTCCTCTCAGCCAATCACAGACGGCCAGCGTCTGTTCTCCATCGCCGTCAGCAGAGTTCAACAT   200
GH-cds Transgenic oyster   .............CCATTCGCAGTGTCTCTGGGTGTTTCCTCTCAGCCAATCACAGACGGCCAGCGTCTGTTCTCCATCGCCGTCAGCAGAGTTCAACAT    87
Consensus                               c  t  g agtgtctctgggtgtttcctctcagccaatcacagacggccagcgtctgttctccatcgccgtcagcagagttcaacat

GH-cds PiggyBac-gGH        CTCCACCTGCTTGCTCAGAGACTCTTCTCCGACTTTGAGAGCACTCTGCAGACGGAGGAGCAGCGACAGCTCAACAAGATCTTCCTGCAGGACTTCTGTA   300
GH-cds Transgenic oyster   CTCCACCTGCTTGCTCAGAGACTCTTCTCCGACTTTGAGAGCACTCTGCAGACGGAGGAGCAGCGACAGCTCAACAAGATCTTCCTGCAGGACTTCTGTA   187
Consensus                  ctccacctgcttgctcagagactcttctccgactttgagagcactctgcagacggaggagcagcgacagctcaacaagatcttcctgcaggacttctgta

GH-cds PiggyBac-gGH        ACTCTGATTACATCATCAGCCCCATCGACAAGCACGAGACGCAGCGCAGCTCCGTGTTGAAGCTGTTGTCGATCTCCTATCGGTTGGTGGAGTCCTGGGA   400
GH-cds Transgenic oyster   ACTCTGATTACATCATCAGCCCCATCGACAAGCACGAGACGCAGCGCAGCTCCGTGTTGAAGCTGTTGTCGATCTCCTATCGGTTGGTGGAGTCCTGGGA   287
Consensus                  actctgattacatcatcagccccatcgacaagcacgagacgcagcgcagctccgtgttgaagctgttgtcgatctcctatcggttggtggagtcctggga

GH-cds PiggyBac-gGH        GTTCCCCAGTCGGTCCCTGTCCGGAGGTTCTGCTCCCAGAAACCAGATTTCTCCCAAACTGTCTGAATTGAAGACCGGGATCCTGCTGCTGATCAGGGCC   500
GH-cds Transgenic oyster   GTTCCCCAGTCGGTCCCTGTCCGGAGGTTCTGCTCCCAGAAACCAGATTTCTCCCAAACTGTCTGAATTGAAGACCGGGATCCTGCTGCTGATCAGGGCC   387
Consensus                  gttccccagtcggtccctgtccggaggttctgctcccagaaaccagatttctcccaaactgtctgaattgaagaccgggatcctgctgctgatcagggcc

GH-cds PiggyBac-gGH        AATCAGGACGGAGCGGAGCTCTTCCCTGACAGCTCCGCCCTCCAGCTGGCTCCTTATGGGAACTATTATCAGAGTCTGGGCGCCGACGAGTCACTGCGAC   600
GH-cds Transgenic oyster   AATCAGGACGGAGCGGAGCTCTTCCCTGACAGCTCCGCCCTCCAGCTGGCTCCTTATGGGAACTATTATCAGAGTCTGGGCGCCGACGAGTCACTGCGAC   487
Consensus                  aatcaggacggagcggagctcttccctgacagctccgccctccagctggctccttatgggaactattatcagagtctgggcgccgacgagtcactgcgac

GH-cds PiggyBac-gGH        GAACGTACGAACTGCTGGCTTGTTTCAAGAAAGACATGCACAAGGTGGAGACCTACCTGACGGTGGCTAAATGTCGACTCTCTCCTGAGGCCAACTGTAC   700
GH-cds Transgenic oyster   GAACGTACGAACTGCTGGCTTGTTTCAAGAAAGACATGCACAAGGTGGAGACCTACCTGACGGTGGCTAAATGTCGACTCTCTCCTGAGGCCAACTGTAC   587
Consensus                  gaacgtacgaactgctggcttgtttcaagaaagacatgcacaaggtggagacctacctgacggtggctaaatgtcgactctctcctgaggccaactgtac

GH-cds PiggyBac-gGH        CCTGTAGGCGGCCGCGAAGGATCTGCGATCGCTCCGGTGCCCGTCAGTGGGCAGAGCGCACATCGCCCACAGTCCCCGAGAAGTTGGGGGGAGGGGTCGG   800
GH-cds Transgenic oyster   CCTGTAGGCGGCCTTAAAAACTATA...........................................................................   612
Consensus                  cctgtaggcggcc   aa   t t
```
