# Supplementary material for: PiggyBac Transposon-Mediated Transgenesis in the Pacific Oyster (Crassostrea gigas) – First Time in Mollusks
Source: Front Physiol. 2018 Jul 16;9:811. doi: 10.3389/fphys.2018.00811 (PMC6054966; doi:10.3389/fphys.2018.00811)
Supplement: FIGURE S3 — Sequence alignment between piggyBac-gGH sequence and GH-GFP segment amplified using the genome of the oyster transfected with piggyBac-gGH as template (PCR amplification using primers GH-GFP-F and GH-GFP-R). The sequence at upstream of the first red arrow is GH segment, that between the two red arrows is the partial plasmid sequence, and that at downstream of the second red arrow is GFP segment. [file Image_3.PDF]

Figure S3.

```
PiggyBac-gGH.                  TCAGAGTCTGGGCGCCGACGAGTCACTGCGACGAACGTACGAACTGCTGGCTTGTTTCAAGAAAGACATGCACAAGGTGGAGACCTACCTGACGGTGGCT   2300
GFP-GH-cds Transgenic oyster   .........................................................................CGGCTGGGTGACTACCTGACGGTGGCT     27
Consensus                                                                                                  g tgg    ctacctgacggtggct

PiggyBac-gGH.                  AAATGTCGACTCTCTCCTGAGGCCAACTGTACCCTGTAGGCGGGCCGCAAGGATCTGCGATCGCTCCGGTGCCCGTCAGTGGGCAGAGCGCACATCGCCC   2400
GFP-GH-cds Transgenic oyster   AA.TGTCGACTCTCTCCTGAGGCCAACTGTACCCTGTAGGCGGCCGCGAAGGATCTGCGATCGCTCCGGTGCCCGTCAGTGGGCAGAGCGCACATCGCCC    126
Consensus                      aa tgtcgactctctcctgaggccaactgtaccctgtaggcgg c   aaggatctgcgatcgctccggtgcccgtcagtgggcagagcgcacatcgccc

PiggyBac-gGH.                  ACAGTCCCCGAGAAGTTGGGGGGAGGGGTCGGCAATTGAACGGGTGCCTAGAGAAGGTGGCGCGGGGTAAACTGGGAAAGTGATGTCGTGTACTGGCTCC   2500
GFP-GH-cds Transgenic oyster   ACAGTCCCCGAGAAGTTGGGGGGAGGGGTCGGCAATTGAACGGGTGCCTAGAGAAGGTGGCGCGGGGTAAACTGGGAAAGTGATGTCGTGTACTGGCTCC    226
Consensus                      acagtccccgagaagttggggggaggggtcggcaattgaacgggtgcctagagaaggtggcgcggggtaaactgggaaagtgatgtcgtgtactggctcc

PiggyBac-gGH.                  GCCTTTTTCCCGAGGGTGGGGGAGAACCGTATATAAGTGCAGTAGTCGCCGTGAACGTTCTTTTTCGCAACGGGTTTGCCGCCAGAACACAGCTGAAGCT   2600
GFP-GH-cds Transgenic oyster   GCCTTTTTCCCGAGGGTGGGGGAGAACCGTATATAAGTGCAGTAGTCGCCGTGAACGTTCTTTTTCGCAACGGGTTTGCCGCCAGAACACAGCTGAAGCT    326
Consensus                      gcctttttcccgagggtgggggagaaccgtatataagtgcagtagtcgccgtgaacgttctttttcgcaacgggtttgccgccagaacacagctgaagct

PiggyBac-gGH.                  TCGAGGGGCTCGCATCTCTCCTTCACGCGCCCGCCGCCCTACCTGAGGCCGCCATCCACGCCGGTTGAGTCGCGTTCTGCCGCCTCCCGCCTGTGGTGCC   2700
GFP-GH-cds Transgenic oyster   TCGAGGGGCTCGCATCTCTCCTTCACGCGCCCGCCGCCCTACCTGAGGCCGCCATCCACGCCGGTTGAGTCGCGTTCTGCCGCCTCCCGCCTGTGGTGCC    426
Consensus                      tcgaggggctcgcatctctccttcacgcgcccgccgccctacctgaggccgccatccacgccggttgagtcgcgttctgccgcctcccgcctgtggtgcc

PiggyBac-gGH.                  TCCTGAACTGCGTCCGCCGTCTAGGTAAGTTTAAAGCTCAGGTCGAGACCGGGCCTTTGTCCGGCGCTCCCTTGGAGCCTACCTAGACTCAGCCGGCTCT   2800
GFP-GH-cds Transgenic oyster   TCCTGAACTGCGTCCGCCGTCTAGGTAAGTTTAAAGCTCAGGTCGAGACCGGGCCTTTGTCCGGCGCTCCCTTGGAGCCTACCTAGACTCAGCCGGCTCT    526
Consensus                      tcctgaactgcgtccgccgtctaggtaagtttaaagctcaggtcgagaccgggcctttgtccggcgctcccttggagcctacctagactcagccggctct

PiggyBac-gGH.                  CCACGCTTTGCCTGACCCTGCTTGCTCAACTCTACGTCTTTGTTTCGTTTTCTGTTCTGCGCCGTTACAGATCCAAGCTGTGACCGGCGCCTACGCTAGA   2900
GFP-GH-cds Transgenic oyster   CCACGCTTTGCCTGACCCTGCTTGCTCAACTCTACGTCTTTGTTTCGTTTTCTGTTCTGCGCCGTTACAGATCCAAGCTGTGACCGGCGCCTACGCTAGA    626
Consensus                      ccacgctttgcctgaccctgcttgctcaactctacgtctttgtttcgttttctgttctgcgccgttacagatccaagctgtgaccggcgcctacgctaga

PiggyBac-gGH.                  CGCCACCATGGAGAGCGACGAGAGCGGCCTGCCCGCCATGGAGATCGAGTGCCGCATCACCGGCACCCTGAACGGCGTGGAGTTCGAGCTGGTGGGCGGC   3000
GFP-GH-cds Transgenic oyster   CGCCACCATGGAGAGCGACGAGAGCGGCCTGCCCGCCATGGAGATCGAGTGCCGCATCACCGGCACCCTGAACGGCGTGGAGTTCGAGCTGGTGGGCGGC    726
Consensus                      cgccaccatggagagcgacgagagcggcctgcccgccatggagatcgagtgccgcatcaccggcaccctgaacggcgtggagttcgagctggtgggcggc

PiggyBac-gGH.                  GGAGAGGGCACCCCCAAGCAGGGCCGCATGACCAACAAGATGAAGAGCACCAAAGGCGCCCTGACCTTCAGCCCCTACCTGCTGAGCCACGTGATGGGCT   3100
GFP-GH-cds Transgenic oyster   GGAGAGGGCACCCCCAAGCAGGGCCGCATGACCAACAAGATGAAGAGCACCAAAGGCGCCCTGACCTTCAGCCCCTACCTGCTGAGCCACGTGATGGGCT    826
Consensus                      ggagagggcacccccaagcagggccgcatgaccaacaagatgaagagcaccaaaggcgccctgaccttcagcccctacctgctgagccacgtgatgggct

PiggyBac-gGH.                  ACGGCTTCTACCACTTCGGCACCTACCCCAGCGGCTACGAGAACCCCTT.CCTGCACGCCATCAACAACGGCGGCTACAC.CAACACCCGCATCGAGAAG   3198
GFP-GH-cds Transgenic oyster   ACGGCTTCTACCACTTCGGCACCTACCCCAGCGGCTACGAGAACCCCTTTCCTGCACGCCATCAACAACGGCGGCTACACACAATGGGGGTGTTGATAGA    926
Consensus                      acggcttctaccacttcggcacctaccccagcggctacgagaacccctt cctgcacgccatcaacaacggcggctacac caa     g  t ga a
```
